# In Inland China, Rice, Rather than Fish, Is the Major Pathway for Methylmercury Exposure

**DOI:** 10.1289/ehp.1001915

**Published:** 2010-04-08

**Authors:** Hua Zhang, Xinbin Feng, Thorjørn Larssen, Guangle Qiu, Rolf D. Vogt

**Affiliations:** 1 State Key Laboratory of Environmental Geochemistry, Institute of Geochemistry, Chinese Academy of Sciences, Guiyang, China; 2 Graduate University of Chinese Academy of Sciences, Beijing, China; 3 Norwegian Institute for Water Research, Oslo, Norway; 4 Department of Chemistry, University of Oslo, Oslo, Norway

**Keywords:** fish, health risk, inland China, methylmercury exposure, rice consumption

## Abstract

**Background:**

Fish consumption is considered the primary pathway of methylmercury (MeHg) exposure for most people in the world. However, in the inland regions of China, most of the residents eat little fish, but they live in areas where a significant amount of mercury (Hg) is present in the environment.

**Objectives:**

We assessed concentrations of total Hg and MeHg in samples of water, air, agricultural products, and other exposure media to determine the main exposure pathway of Hg in populations in inland China.

**Methods:**

We selected Guizhou Province for our study because it is highly contaminated with Hg and therefore is representative of other Hg-contaminated areas in China. We selected four study locations in Guizhou Province: three that represent typical environments with severe Hg pollution [due to Hg mining and smelting (Wanshan), traditional zinc smelting (recently closed; Weining), and heavy coal-based industry (Qingzhen)], and a village in a remote nature reserve (Leigong).

**Results:**

The probable daily intake (PDI) of MeHg for an adult population based on 60 kg body weight (bw) was considerably higher in Wanshan than in the other three locations. With an average PDI of 0.096 μg/kg bw/day (range, 0.015–0.45 μg/kg bw/day), approximately 34% of the inhabitants in Wanshan exceeded the reference dose of 0.1 μg/kg bw/day established by the U.S. Environmental Protection Agency. The PDI of MeHg for residents in the three other locations were all well below 0.1 μg/kg bw/day (averages from 0.017 to 0.023 μg/kg bw/day, with a maximum of 0.095 μg/kg bw/day). In all four areas, rice consumption accounted for 94–96% of the PDI of MeHg.

**Conclusion:**

We found that rice consumption is by far the most important MeHg exposure route; however, most of the residents (except those in Hg-mining areas) have low PDIs of MeHg.

All over the world, mercury (Hg) is present in various environmental media and foods (especially fish) at levels that can adversely affect humans and wildlife. Methylmercury (MeHg), one of the most toxic of the organic Hg forms, is readily bioavailable and biomagnifies in the food chain so that fish at higher trophic levels regularly have Hg concentrations a million-fold higher than the water in which they live (Stein et al. 1996). Of MeHg consumed, 95%, on average, is absorbed [World Health Organization (WHO) 1990]. Hg has a detrimental effect on the central nervous system because it can easily cross the blood–brain barrier and placental barriers. Consumption of food contaminated with MeHg has resulted in several endemic disasters in Japan, Iraq, and elsewhere in the last century (Harada 1995). Today, fish consumption is considered the only significant source of dietary exposure to MeHg for humans (Mergler et al. 2007).

Situated in the center of the circum-Pacific mercuriferous belt, Guizhou is known as the “mercury capital” of China (Qiu et al. 2005). The total reserves of cinnabar deposits in the province have reached 80,000 metric tons of metal Hg and represent 80% of the total Hg in China (Feng and Qiu 2008). At least 12 large Hg mines have been operating in the province, making Guizhou one of the world’s largest Hg production centers.

Coal combustion and zinc (Zn) smelting are also important sources of anthropogenic Hg in Guizhou. Approximately 80% of total energy demand in the province is provided by coal combustion (Feng and Qiu 2008). The Hg concentration in local coal is higher than in coal from other provinces of China (Feng et al. 2002); therefore, large quantities of Hg are also released to the environment from coal combustion in Guizhou, resulting in widespread Hg contamination to the ambient air and local environment.

Artisanal (small-scale) Zn-smelting activities (using an indigenous method) have been ongoing for at least a century in Guizhou, with a large number of smelters scattered throughout the northwestern parts of the province (i.e., Hezhang and Weining). Because of the geological background of the mercuriferous belt, significant amounts of Hg have been found in Zn sulfide ores from these areas. During Zn-smelting processes, Hg^2+^ is reduced to elementary Hg and evaporated. A large quantity of Hg is thereby released to the environment, resulting in additional serious Hg contamination to the local environment (Feng et al. 2004a).

All of these large anthropogenic Hg emissions sources, in addition to a general elevated natural background level, result in much higher amounts of Hg in the environment, even in pristine regions of Guizhou, compared with undisturbed regions in North America and Europe (Fu et al. 2009). Under certain conditions that promote the activity of Hg-methylating bacteria, the Hg may become methylated, leading to increased bioavailability of Hg, followed by bioaccumulation in the food chain (Ullrich et al. 2001). For instance, rice paddy soil has been proven to be a suitable environment for sulfur-reducing bacteria (Stubner et al. 1998) and favorable for Hg methylation processes. Furthermore, Krupp et al. (2009) recently found that phytochelatins, small peptides that detoxify heavy metals in rice plants, can sequester Hg^2+^ but not MeHg. These observations suggest that MeHg produced in the paddy soil might be easily taken up in the rice plant.

Poorer segments of the population are less able to avoid exposure to pollutants and thereby protect themselves from elevated Hg pollution. Guizhou is an undeveloped region in inland China, and for rural households in this area, the annual per capita net income is only 1,985 CNY (Chinese yuan; ~ US$290). More than 27 million people live in the rural areas of Guizhou, accounting for 72.5% of its total population [Bureau of Guizhou Statistics (BGS) 2007].

Serious Hg contamination has been reported in different environmental media in Guizhou, including elevated Hg^0^ concentrations up to 1,100 ng/m^3^ in ambient air (Qiu et al. 2005; Wang et al. 2007), total mercury (THg) up to 12,000 ng/L in surface water samples (Horvat et al. 2003; Zhang et al. 2010), and 790 mg/kg in paddy soil samples (Horvat et al. 2003; Qiu et al. 2005) from Hg-mined areas. However, an assessment of THg and MeHg exposure to the population has been lacking. A recent study by Li et al. (2009) showed that fish in Guizhou contain low levels of THg, with an average concentration of only 0.063 mg/kg (*n* = 228), with only one fish exceeding 0.5 mg/kg, the maximum MeHg limit for fish set by the Standardization Administration of the People’s Republic of China (SAC 2005). Furthermore, the residents of Guizhou rarely eat fish [only 1.2 g/day/person according to the Bureau of Guizhou Statistics (2007)]. This result appears to indicate that the population in Guizhou may not have substantial MeHg exposure. However, studies in Hg-mining areas in Guizhou found elevated MeHg concentration in agricultural products. For instance, Horvat et al. (2003) observed MeHg concentrations in rice as high as 140 μg/kg, whereas Qiu et al. (2008) reported levels of 170 μg/kg in Wanshan. In a follow-up study, Feng et al. (2008) observed high MeHg concentrations in human hair samples collected from three villages in Wanshan (averages from 1.3 to 2.8 mg/kg, with a maximum of 5.6 mg/kg); these concentrations were positively correlated with calculated MeHg exposure doses via food consumption (*R*^2^ = 0.42; *p* < 0.001).

In the present study, we selected four locations representing typical rural areas in Guizhou province where inhabitants eat mainly local agricultural products they have planted themselves. The four areas are Wanshan (representing areas impacted by Hg-mining and smelting activities), Qingzhen (representing areas impacted by a coal-fired power plant), Weining (representing areas affected by historical artisanal Zn-smelting activities), and Leigong Natural Reserve (representing areas with no direct Hg contamination sources) (Figure 1). We assessed the important MeHg exposure pathways via drinking water, diet (fish, rice, corn, vegetables, meat, and poultry), and respiration and to evaluate their potential health impacts in the general adult population of Guizhou in order to provide new understanding of MeHg exposure pathways for populations with low fish consumption and to help the local governments and health agencies to develop intervention policies and education strategies to protect populations from overexposure to MeHg.

## Materials and Methods

### Sample collection

**Figure 1:**
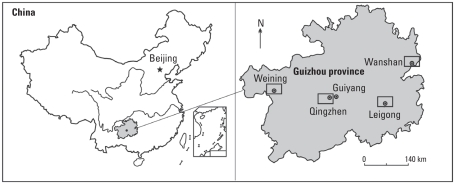
Locations of four research areas in Guizhou, China. For details of detailed sampling locations, see Supplemental Material, Figures 1–4 (doi:10.1289/ehp.1001915).

Building on previous published data from the four selected locations (Figure 1) for air (Feng et al. 2004b; Fu et al. 2009), water (Feng et al. 2004a, 2004b; He et al. 2008), fish (Li et al. 2009; Qiu et al. 2009), meat (Feng et al. 2008), and poultry (Ji et al. 2006) (Table 1), we expanded the scope by conducting supplementary sampling over a larger area, covering > 700 km^2^ in both Wanshan and Qingzhen areas. We collected samples of agricultural products (i.e., rice, corn, vegetables) directly from the fields, and drinking water samples (water samples collected only in Wanshan and Leigong) from domestic wells and reservoirs for human consumption. In Wanshan, we also measured total gaseous mercury (TGM) *in situ*. Sampling was carried out during September 2007 in Wanshan and Qingzhen, during August 2008 in Weining, and during September 2008 in Leigong. Data from the present study and from the literature are summarized in Table 1. For detailed methods of sample collection and preparation, see Supplemental Material, Figures 1–4 and Section 1 (doi:10.1289/ehp.1001915).

### Analytical methods

The edible part of the grain (rice and corn) and vegetable samples were dried in an oven at 40°C until they reached constant weight. We then crushed the edible part of the samples and sieved them through a 150-mesh sieve. For THg analysis, the sieved samples (0.1–0.2 g) were digested at 95°C with a fresh mixture of nitric acid/sulfuric acid (vol/vol 4:1) (Feng et al. 2008). Concentrations of THg in the samples were determined by cold vapor atomic fluorescence spectroscopy (CVAFS) after bromine chloride oxidation, stannous chloride reduction, purging, and up-concentrating by gold trapping using Method 1631 [U.S. Environmental Protection Agency (EPA) 2002]. For MeHg analysis, the sieved samples (0.1–0.2 g) were digested using the potassium hydroxide-methanol/solvent extraction technique (Liang et al. 1996). We measured MeHg contents in these samples using gas chromatography (GC)-CVAFS detection after aqueous ethylation, purging, and trapping (Liang et al. 1994) using Method 1630 (U.S. EPA 2001a). Within 28 days after sampling, we analyzed concentrations of THg in water samples using the dual-stage gold amalgamation method and CVAFS detection according to U.S. EPA Method 1631 (U.S. EPA 2002). MeHg in water samples was analyzed by GC-CVAFS detection after distillation and ethylation using U.S. EPA Method 1630 (U.S. EPA 2001a). Measurements of TGM in ambient air were performed using a portable Zeeman Mercury Analyzer RA-915+ (Lumex Ltd., St. Petersburg, Russia). Information on quality assurance and quality control of our measurement data is available in Supplemental Material, Section 2 (doi:10.1289/ehp.1001915).

### Calculation of probable daily intake (PDI)

To determine MeHg and THg exposure via drinking water, inhalation, and food consumption, we calculated PDI values for the general adult population according to the following formula:


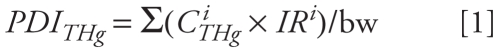



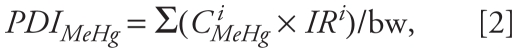


where PDI is given in micrograms per kilogram of body weight (bw) per day; bw = 60 kg; *C* is the concentration of exposed medium; *IR* is intake rate (or ingestion rate or inhalation rate), and *i* = intake of air, water, rice, fish, vegetable, corn, meat, and poultry.

This calculation is based on the assumption that MeHg exposure from other routes [i.e., ambient atmosphere (Gnamus et al. 2000; WHO 1990); dental amalgam fillings (Barregard et al. 1995; Batista et al. 1996); other foods (i.e., food oil, salt, beverage such as milk) (Cheng et al. 2009); and dermal exposure (U.S. EPA 1997; WHO 2003)] is negligible.

The intake rates for different exposure media for the adult populations used were based on the Guizhou Statistical Yearbook reported by BGS (2007) (Table 2).

To better relate the different characteristics of Hg exposure in the population in inland China who consume a a rice-based diet with those of a population who consume more fish in their diet, we used two typical regions with high fish consumption for comparison: a Japanese population of rural, coastal women (Iwasaki et al. 2003), and a reference group of the general Norwegian population (Mangerud 2005). We also compared the MeHg exposure in the present study with the MeHg exposure assessment of women in the U.S. general population (Carrington and Bolger 2002; Mahaffey et al. 2004). These calculations were based on the assumption that each adult’s body weight was 60 kg for the Guizhou population and for U.S. women, 55 kg for Japanese women, and 70 kg for the Norwegian population.

### Statistical methods

Because concentrations of Hg in the environment in Wanshan varied greatly with distance from the pollution source, site-specific exposure assessments were conducted based on samples of food collected at 59 selected sites to reflect the regional difference. For the other three locations (Qingzhen, Weining, and Leigong), the calculations were based only on the mean, minimum, and maximum concentrations of different media because of the generally relatively low concentrations and the small SD.

## Results

### Hg levels in different exposure media

In general, Hg concentrations in all exposure media in Qingzhen, Weining, and Leigong were well below the corresponding Chinese national standard limit (Table 1). However, we found elevated average Hg concentrations in samples from Wanshan (rice, 78 μg/kg for THg and 9.3 μg/kg for MeHg; vegetables, 130 μg/kg for THg and 0.097 μg/kg for MeHg; meat, 220 μg/kg for THg and 0.85 μg/kg for MeHg; poultry, 160 μg/kg for THg and 2.4 μg/kg for MeHg; air, 93 ng/m^3^ for TGM). In all four locations, fish contained low average concentrations of Hg (THg, 0.29 mg/kg in Wanshan and 0.063 mg/kg in the other three locations; MeHg, 0.060 mg/kg in Wanshan and 0.014 mg/kg in the other locations), well below the Chinese national guideline of 0.5 mg/kg for MeHg (Table 1).

### PDI levels

**Figure 2:**
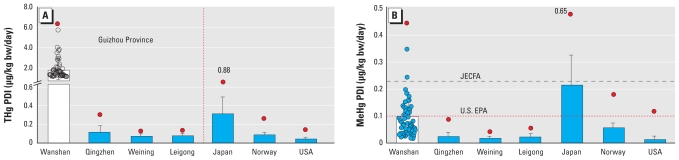
Calculated PDI (mean ± SD) of THg (*A*) and MeHg (*B*) for the adult population in Guizhou (primarily rice-based diet) and for adults in Japan, Norway, and the United States who ate a more fish-based diet. The data for Japan are from a population of rural, coastal women (Iwasaki et al. 2003); the Norwegian data are from a reference group of the general population (Mangerud 2005); and the U.S. data are from women in the general population (Carrington and Bolger 2002; Mahaffey et al. 2004). The black dashed line represents the PTWI of 0.23 μg/kg/day recommended by JECFA (2003), and the red dotted line indicates the U.S. EPA RfD of 0.10 μg/kg/day (U.S. EPA 2001b). In (*A*) and (*B*), the red circles represent the maximum value; open circles (*A*) and blue circles (*B*) represent values for individuals in the Wanshan area.

The calculated average of the PDI of THg for the adult population in Wanshan was 1.9 μg/kg bw/day (range, 0.25–6.4 μg/kg bw/day). This was significantly higher (*p* < 0.01) than the values obtained from the other three locations, which were 0.11, 0.069, and 0.075 μg/kg bw/day for Qingzhen, Weining, and Leigong, respectively (Figure 2A). For MeHg, the PDI was also significantly higher (*p* < 0.01) in Wanshan (average, 0.096 μg/kg bw/day) than in the other three locations (averages of 0.017–0.023 μg/kg bw/day) (Figure 2B).

### Contributions to Hg exposure from different media

**Figure 3:**
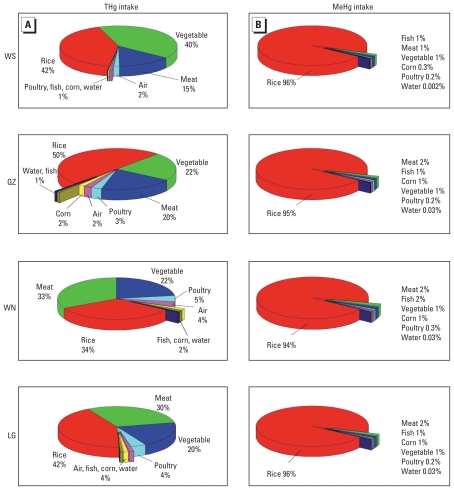
Percentage of estimated THg intake (*A*) and MeHg intake (*B*) from different media for the adult populations in Guizhou. Abbreviations: LG, Leigong; QZ, Qingzhen; WN, Weining; WS, Wanshan.

Consumption of rice, vegetables, and meat, as a whole, accounted for > 90% of the PDI of THg (Figure 3A). Rice contributed 34–50%, vegetables 22–42%, and meat 15–33% in whole study areas. Fish, ambient air, poultry, corn, and drinking water accounted for only a small part of the total daily intake. For the PDI of MeHg, rice consumption is by far the largest source in all of the areas, accounting for between 94% and 96% of total MeHg intake (Figure 3B).

### Risk considerations

The PDI of THg for adult populations in Qingzhen, Weining, and Leigong (means of 0.068–0.11 μg/kg bw/day; maximum of 0.31 μg/kg bw/day) were all well below the provisional tolerable weekly intake (PTWI) of 4 μg/kg bw/week (equal to 0.57 μg/kg bw/day) [Joint FAO/WHO Expert Committee on Food Additives (JECFA) 2010]. However, the PDI of THg for adult populations in all selected sites in Wanshan greatly exceeded 0.57 μg/kg bw/day (Figure 2A), suggesting a potential health risk to local inhabitants.

Similarly, the PDI of MeHg for adult populations was also considerably higher in Wanshan than in the three other areas. With an average PDI of 0.096 μg/kg bw/day (range, 0.015–0.45 μg/kg bw/day), approximately 7% of adult inhabitants in the 59 selected sites in Wanshan exceeded the new PTWI for MeHg of 1.6 μg/kg bw/week (equivalent to 0.23 μg/kg bw/day) (JECFA 2003), whereas 34% exceeded the reference dose (RfD) of 0.1 μg/kg bw/day (U.S. EPA 2001b). The PDI of MeHg for adult residents in the three other locations (Qingzhen, Weining, and Leigong) were all well below the strictest RfD of 0.1 μg/kg bw/day (averages of 0.017–0.023; maximum of 0.095 μg/kg bw/day) (Figure 2B).

## Discussion

### General characteristics of Hg exposure

Our results show that rice is by far the most important source of MeHg in the four locations (94–96%; Figure 3B). For THg, vegetables and meat also contribute considerably (Figure 3A), but these food items have very low MeHg concentrations (Table 1). Because of low fish consumption (1.2 g/day/person) (BGS 2007) and low Hg concentrations (Table 1), the contribution of fish to the Hg intake is low (1–2%). This result was completely different in studies in other countries where fish is usually the dominant source of Hg (Mergler et al. 2007). Rice, the predominant dietary food staple for the Guizhou population (600 g/day/person) (BGS 2007; Qiu et al. 2008), contains relatively higher MeHg levels compared with other crops because of its growing conditions in water-saturated soils, with reducing conditions and a favorable environment for Hg methylation (Qiu et al. 2005; Stubner et al. 1998).

As shown in Figure 2A, the PDI of THg for adult inhabitants in Wanshan [1.9 (range, 0.25–6.4) μg/kg bw/day] was much higher than the PDIs for two populations with a high-fish diet: a population of rural, coastal women in Japan [0.31 (range, 0.037–0.88) μg/kg bw/day] (Iwasaki et al. 2003) and a reference group of adults in the general Norwegian population [0.077 (range, 0.037–0.24) μg/kg bw/day] (Mangerud 2005). The PDI for THg for adult populations in Qingzhen, Weining, and Leigong (averages of 0.069–0.11 μg/kg bw/day) was similar to that of the adult Norwegian reference group.

Conversely, the PDI of MeHg for Wanshan [0.096 (range, 0.015–0.45) μg/kg bw/day] was much lower than that for Japanese women who consumed a high-fish diet [0.21 (range, 0.037–0.65) μg/kg bw/day] (Iwasaki et al. 2003), despite the fact that the adults in Wanshan have a much higher THg PDI. Still, the Wanshan population has a higher MeHg PDI relative to the Norwegian reference group [0.058 (range, 0.028–0.18) μg/kg bw/day] (Mangerud 2005). Similarly, the PDIs of MeHg for adult populations in Qingzhen, Weining, and Leigong (averages of 0.017–0.023 μg/kg bw/day) were lower than those for the Norwegian reference group, although they have similar THg PDIs. The PDIs of MeHg in Qingzhen, Weining, and Leigong were close to those of the U.S. adult women [0.013 μg/kg bw/day (Carrington and Bolger 2002) or 0.02 μg/kg bw/day (Mahaffey et al. 2004)].

The average ratio of the MeHg PDI to the THg PDI for the population of Guizhou was 5–29% (Table 2), which differs from that in Japan, Norway, the United States, and other countries and regions (generally reaching 75–99%) (Iwasaki et al. 2003; Mahaffey et al. 2004; Mangerud 2005; U.S. EPA 1997).

The adult population of Wanshan has a much lower MeHg exposure (Figure 2B) but a much higher THg dose than the Japanese and Norwegian adult populations. This may be due to the fact that the Japanese and Norwegian adult populations were exposed to Hg mainly through fish consumption, where 75–95% of the Hg is MeHg (Bloom 1992; Iwasaki et al. 2003; Mangerud 2005), whereas in the foodstuffs in Wanshan, about 95% of the Hg is in the inorganic form (e.g., about 75% in fish, 80% in rice, 99.9% in vegetables, and 98% in meat).

As noted above, we observed that Hg concentrations in fish were very low not only from environments devoid of direct contamination (Li et al. 2009) but also from Hg-mined areas (Qiu et al. 2009); hence, fish consumption is a minor contributor to MeHg exposure in Guizhou. Many of the commonly eaten fish species in inland China are fast-growing species, often herbivorous or omnivorous with a short food chain, that will not accumulate much Hg. In addition, most commonly eaten fish are farmed fish, typically fast-growing and fed on vegetable-based fodder. However, fish consumption is still a major source of MeHg intake in some coastal areas of China where fish contain elevated MeHg concentrations and residents have high consumption of fish (Cheng et al. 2009).

The contrast of THg and MeHg exposure between the Guizhou population and the Japanese and Norwegian populations, as shown in Figure 2, suggests that the PDI of THg should not be used to evaluate Hg exposure in populations with a rice-based diet, such as in Guizhou. A considerable amount of the Hg in food in Guizhou was inorganic Hg, which is much less toxic than MeHg (Clarkson and Magos 2006). Furthermore, the absorption rate for Hg^2+^ by the human body through food consumption has been estimated to be only 7% (Clarkson and Magos 2006; WHO 1991), whereas 95% of MeHg is assimilated (WHO 1990). Instead, a PDI based on levels of MeHg in rice should be used for evaluation of Hg exposure for the population in Guizhou, based on diet.

### Food consumption advisories

Hg concentrations in fish in Guizhou were below the limit set by Chinese authorities (0.5 mg/kg) (SAC 2005), with only a few exceptions. In Guizhou Hg exposure through fish consumption does not appear to be of particular concern, and consumption advisories are not required. These results are in stark contrast to the situation in high fish-consuming regions in Japan, North America, and northern Europe, where (wild) fish may contain considerably higher MeHg concentrations than recommended values and fish consumption in those regions is generally high (possibly up to 200 g/day/person) (Canuel et al. 2006; Iwasaki et al. 2003; Mangerud 2005).

Because rural residents in Guizhou rarely eat fish, MeHg exposure is mainly through rice consumption. Thus, for an adult who consumes 600 g of rice daily, according to the RfD of 0.1 μg/kg bw/day (U.S. EPA 2001b), the limit of MeHg is 10 μg/kg rice. This value should be used as the tolerable concentration for MeHg in rice where rice is the dietary staple for the population. This value is consistent with the standard limit for THg concentration in rice recommended by SAC (2005) (i.e., 20 μg/kg for food other than fish) if MeHg is 50% of THg. For the highly contaminated rice observed in this study (maximum MeHg concentration of 44 μg/kg), the maximum daily intake of rice should be ≤ 130 g for adults with a body weight of 60 kg to avoid exceeding the daily RfD of 0.1 μg/kg established by the U.S. EPA (2001b).

Rice does not contain the same important micronutrients associated with fish, such as docosahexaenoic acid (DHA, an omega-3 long-chain polyunsaturated fatty acid), arachidonic acid (an omega-6), and iodine, all of which enhance neurodevelopment (Budtz-Jørgensen et al. 2007; Jacobson et al. 2008). Because people in Guizhou consume a rice-based diet, the MeHg RfD based on fish consumption may be inadequate to protect the population from adverse effects from Hg exposure. Research on the health impacts should be conducted in the future, especially regarding pregnant women in inland China who have been exposed to low doses of MeHg through consumption of rice. Furthermore, whether there is a synergetic effect on human health with coexposure of MeHg and inorganic Hg is still unknown.

### Percentage of total population under potential health risk

In addition to Wanshan, there are 11 other Hg mining and smelting areas in Guizhou (Feng and Qiu 2008), with populations totaling approximately 320,000 (BGS 2007). Approximately 22,400 residents in Guizhou (0.06% of the total population) are exposed to Hg concentrations of ≥ 0.23 μg/kg bw/day, and approximately 107,200 residents (0.28% of the total population) are exposed to ≥ 0.1 μg/kg bw/day (BGS 2007). These estimates were based on the assumption that these populations are exposed to MeHg at concentrations similar to those found in Wanshan; that is, 7% of inhabitants living near the Hg mines were exposed to ≥ 0.23 μg/kg bw/day and 34% were exposed to ≥ 0.1 μg/kg bw/day.

Actually, rice is the staple food of more than half the world’s population [Food and Agriculture Organization of the United Nations (FAO) 2006]. In Asia alone, > 2 billion people get up to 70% of their daily dietary energy from rice and its by-products (FAO 2006). Related research is urgently needed not only in China but also in other countries and regions (e.g., India, Indonesia, Bangladesh, the Philippines) that produce a significant percentage of the global rice crops and where rice is a staple food (International Rice Research Institute 2009). In some countries, extensive Hg contamination has already been well documented [e.g., from Hg mining in the Philippines (Gray et al. 2003) and industrial pollution in India (Sharma 2003)].

## Conclusions

In the present study we found that the general adult population in Guizhou is exposed to low levels of MeHg that may not pose serious health risks to most of the population. Nevertheless, in a small portion of the population in heavily contaminated Hg-mining areas, MeHg exposure may exceed the tolerable intake for pregnant women. Rice consumption is the predominant pathway of MeHg exposure to the general population of Guizhou. Moreover, fish consumption contributes only 1–2% of their MeHg exposure, which is much lower than reported in Japan, North America, and Europe. However, inhabitants in Hg-mining areas were exposed to high levels of both MeHg and inorganic Hg. Studies are needed to determine whether dose–response relationships established for MeHg through fish and seafood consumption is valid for populations exposed through rice consumption.

## Figures and Tables

**Table 1:** Average concentrations of THg and MeHg [and corresponding Chinese national limits (SAC 2005)] of all main exposure media in Guizhou.

	THg					MeHg				
Medium	WS	QZ	WN	LG	Limit	WS	QZ	WN	LG	Limit
Air (ng/m^3^)	93^a^	7.5^b^	7.5^b^,^c^	2.8^d^						
Water (ng/L)	50^a^	19^b^,^e^	13^f^	1.5^a^	1,000	0.064^a^	0.22^b^,^e^	0.13^f^	0.047^a^	
Rice (μg/kg DW)	78^a^	5.5^a^	2.3^a^	3.2^a^	20	9.3^a^	2.2^a^	1.6^a^	2.1^a^	
Corn (μg/kg DW)	2.3^a^	1.9^a^	0.71^a^	0.59^a^	20	0.25^a^	0.21^a^	0.15^a^	0.13^a^	
Fish (μg/kg WW)	290^g^	66^h^	66^h^	66^h^		60^g^	14 c,h	14^c^,^h^	14^c^,^h^	500
Vegetables (μg/kg WW)	130^a^,^i^	4.0^a^	2.5^a^	2.5^a^	10	0.097^a^,^i^	0.032^a^	0.023^a^	2.5^a^	
Meat (μg/kg WW)	220^i^	17^c^,^j^	17^c^,^j^	17^c^,^j^	50	0.85^i^	0.26^c^,^j^	0.26^c^,^j^	0.26^c^,^j^	
Poultry (μg/kg WW)	160^k^	39^c^,^j^	39^c^,^j^	39^c^,^j^		2.4^k^	0.56^c^,^j^	0.56^c^,^j^	0.56^c^,^j^	

Abbreviations: DW, dry weight; LG, Leigong; QZ, Qingzhen; WN, Weining; WS, Wanshan; WW, wet weight. More detailed information is available in Supplemental Material, Tables 1–7 (doi:10.1289/ehp.1001915).

a Present study.

b Feng et al. (2004b).

c Estimated value.

d Fu et al. (2009).

e He et al. (2008).

f Feng et al. (2004a).

g Qiu et al. (2009).

h Li et al. (2009).

i Feng et al. (2008).

j Cheng et al. (2009).

k Ji et al. (2006).

**Table 2:** Average estimated daily intake of THg and MeHg through all main routes for adults (60 kg bw) in the rural population of different areas of Guizhou.

		THg daily intake (μg/day)				MeHg daily intake (μg/day)			
Medium	Intake rate	WS	QZ	WN	LG	WS	QZ	WN	LG
Air	20 m^3^/day	1.9	0.15	0.15	0.056	a	a	a	a
Water	2 L/day	0.10	0.038	0.026	0.0030	0.00013	0.00044	0.00026	0.000094
Rice	600 g/day	49	3.3	1.4	1.9	5.6	1.3	0.96	1.3
Corn	60 g/day	0.11	0.11	0.043	0.035	0.015	0.013	0.0090	0.0078
Vegetable	368 g/day	47	1.5	0.92	0.92	0.036	0.012	0.0085	0.0085
Meat	79.3 g/day	17	1.4	1.4	1.4	0.067	0.021	0.021	0.021
Fish	1.2 g/day	0.35	0.054	0.054	0.054	0.073	0.017	0.017	0.017
Poultry	4.9 g/day	0.77	0.19	0.19	0.19	0.011	0.0026	0.0026	0.0026
Total	μg/day	116	6.7	4.1	4.5	5.8	1.4	1.0	1.3
	μg/kg/day	1.9	0.11	0.069	0.075	0.096	0.023	0.017	0.022
MeHg PDI/THg PDI						5%	21%	24%	29%

Abbreviations: LG, Leigong; QZ, Qingzhen; WN, Weining; WS, Wanshan.

a Negligible.

## References

[b1-ehp-118-1183] Barregard L, Sallsten G, Jarvholm B (1995). People with high mercury uptake from their own dental amalgam fillings. Occup Environ Med.

[b2-ehp-118-1183] Batista J, Schuhmacher M, Domingo JL, Corbella J (1996). Mercury in hair for a child population from Tarragona Province, Spain. Sci Total Environ.

[b3-ehp-118-1183] BGS (Bureau of Guizhou Statistics) (2007). Guizhou Statistical Yearbook 2006.

[b4-ehp-118-1183] Bloom NS (1992). On the chemical form of mercury in edible fish and marine invertebrate tissue. Can J Fish Aquat Sci.

[b5-ehp-118-1183] Budtz-J⊘rgensen E, Grandjean P, Weihe P (2007). Separation of risks and benefits of seafood intake. Environ Health Perspect.

[b6-ehp-118-1183] Canuel R, de Grosbois SB, Atikessé L, Lucotte M, Arp P, Ritchie C (2006). New evidence on variations of human body burden of methylmercury from fish consumption. Environ Health Perspect.

[b7-ehp-118-1183] Carrington CD, Bolger MP (2002). An exposure assessment for methylmercury from seafood for consumers in the United States. Risk Anal.

[b8-ehp-118-1183] Cheng JP, Gao LL, Zhao WC, Liu XJ, Sakamoto M, Wang WH (2009). Mercury levels in fisherman and their household members in Zhoushan, China: impact of public health. Sci Total Environ.

[b9-ehp-118-1183] Clarkson TW, Magos L (2006). The toxicology of mercury and its chemical compounds. Crit Rev Toxicol.

[b10-ehp-118-1183] FAO (Food and Agriculture Organization of the United Nations) (2006). “Rice Is Life”: International Rice Commission Meets in Peru.

[b11-ehp-118-1183] Feng XB, Li GH, Qiu GL (2004a). A preliminary study on mercury contamination to the environment from artisanal zinc smelting using indigenous methods in Hezhang county, Guizhou, China. Part 1. Mercury emission from zinc smelting and its influences on the surface waters. Atmos Environ.

[b12-ehp-118-1183] Feng XB, Li P, Qiu GL, Wang S, Li GH, Shang LH (2008). Human exposure to methylmercury through rice intake in mercury mining areas, Guizhou Province, China. Environ Sci Technol.

[b13-ehp-118-1183] Feng XB, Qiu GL (2008). Mercury pollution in Guizhou, Southwestern China—an overview. Sci Total Environ.

[b14-ehp-118-1183] Feng XB, Sommar J, Lindqvist O, Hong YT (2002). Occurrence, emissions and deposition of mercury during coal combustion in the Province Guizhou, China. Water Air Soil Pollut.

[b15-ehp-118-1183] Feng XB, Yan HY, Wang SF, Qiu GL, Tang SL, Shang LH (2004b). Seasonal variation of gaseous mercury exchange rate between air and water surface over Baihua reservoir, Guizhou, China. Atmos Environ.

[b16-ehp-118-1183] Fu XW, Feng X, Dong ZQ, Yin RS, Wang JX, Yang ZR (2009). Atmospheric total gaseous mercury (TGM) concentrations and wet and dry deposition of mercury at a high-altitude mountain peak in south China. Atmos Chem Phys Discuss.

[b17-ehp-118-1183] Gnamus A, Byrne AR, Horvat M (2000). Mercury in the soil-plant-deer-predator food chain of a temperate forest in Slovenia. Environ Sci Technol.

[b18-ehp-118-1183] Gray JE, Greaves IA, Bustos DM, Krabbenhoft DP (2003). Mercury and methylmercury contents in mine-waste calcine, water, and sediment collected from the Palawan Quicksilver Mine, Philippines. Environ Geol.

[b19-ehp-118-1183] Harada M (1995). Minamata disease: methylmercury poisoning in Japan caused by environmental pollution. Crit Rev Toxicol.

[b20-ehp-118-1183] He TR, Feng XB, Guo YN, Qiu GL, Li ZG, Liang L (2008). The impact of eutrophication on the biogeochemical cycling of mercury species in a reservoir: a case study from Hongfeng Reservoir, Guizhou, China. Environ Pollut.

[b21-ehp-118-1183] Horvat M, Nolde N, Fajon V, Jereb V, Logar M, Lojen S (2003). Total mercury, methylmercury and selenium in mercury polluted areas in the province Guizhou, China. Sci Total Environ.

[b22-ehp-118-1183] International Rice Research Institute (2009). http://beta.irri.org/solutions/index.php?option=com_content&task=view&id=250.

[b23-ehp-118-1183] Iwasaki Y, Sakamoto M, Nakai K, Oka T, Dakeishi M, Iwata T (2003). Estimation of daily mercury intake from seafood in Japanese women: Akita cross-sectional study. Tohoku J Exp Med.

[b24-ehp-118-1183] Jacobson JL, Jacobson SW, Muckle G, Kaplan-Estrin M, Ayotte P, Dewailly E (2008). Beneficial effects of a polyunsaturated fatty acid on infant development: evidence from the Inuit of arctic Quebec. J Pediatr.

[b25-ehp-118-1183] JECFA (Joint FAO/WHO Expert Committee on Food Additives) (2003). https://apps.who.int/pcs/jecfa/Summary61.pdf.

[b26-ehp-118-1183] JECFA (Joint FAO/WHO Expert Committee on Food Additives) (2010). Joint FAO/WHO Food Standards Programme, Committee of the Codex Alimentarius Commission, Thirty-third Session.

[b27-ehp-118-1183] Ji X, Hu W, Cheng J, Yuan T, Xu F, Qu LY (2006). Oxidative stress on domestic ducks (*Shaoxing duck*) chronically exposed in a mercury-selenium coexisting mining area in China. Ecotox Environ Safe.

[b28-ehp-118-1183] Krupp EM, Mestrot A, Wielgus J, Meharg AA, Feldmann J (2009). The molecular form of mercury in biota: identification of novel mercury peptide complexes in plants. Chem Commun.

[b29-ehp-118-1183] Li SX, Zhou LF, Wang HJ, Liang YG, Chang JB (2009). Feeding habits and habitats preferences affecting mercury bioaccumulation in 37 subtropical fish species from Wujiang River, China. Ecotoxicology.

[b30-ehp-118-1183] Liang L, Horvat M, Bloom NS (1994). An improved speciation method for mercury by GC/CVAFS after aqueous phase ethylation and room temperature precollection. Talanta.

[b31-ehp-118-1183] Liang L, Horvat M, Cernichiari E, Gelein B, Balogh S (1996). Simple solvent extraction technique for elimination of matrix interferences in the determination of methylmercury in environmental and biological samples by ethylation gas chromatography cold vapor atomic fluorescence spectrometry. Talanta.

[b32-ehp-118-1183] Mahaffey KR, Clickner RP, Bodurow CC (2004). Blood organic mercury and dietary mercury intake: National Health and Nutrition Examination Survey, 1999 and 2000. Environ Health Perspect.

[b33-ehp-118-1183] Mangerud G (2005). Dietary Mercury Exposure in Selected Norwegian Municipalities. The Norwegian Fish and Game Study.

[b34-ehp-118-1183] Mergler D, Anderson HA, Chan LHM, Mahaffey KR, Murray M, Sakamoto M (2007). Methylmercury exposure and health effects in humans: a worldwide concern. Ambio.

[b35-ehp-118-1183] Qiu GL, Feng XB, Li P, Wang SF, Li GH, Shang LH (2008). Methylmercury accumulation in rice (*Oryza sativa* L.) grown at abandoned mercury mines in Guizhou, China. J Agric Food Chem.

[b36-ehp-118-1183] Qiu GL, Feng XB, Wang SF, Fu XW, Shang LH (2009). Mercury distribution and speciation in water and fish from abandoned Hg mines in Wanshan, Guizhou province, China. Sci Total Environ.

[b37-ehp-118-1183] Qiu GL, Feng XB, Wang SF, Shang LH (2005). Mercury and methylmercury in riparian soil, sediments, mine-waste calcines, and moss from abandoned Hg mines in east Guizhou Province, southwestern China. Appl Geochem.

[b38-ehp-118-1183] SAC (Standardization Administration of the People’s Republic of China) (2005). Maximum Levels of Contaminants in Foods [in Chinese].

[b39-ehp-118-1183] Sharma DC (2003). Concern over mercury pollution in India. Lancet.

[b40-ehp-118-1183] Stubner S, Wind T, Conrad R (1998). Sulfur oxidation in rice field soil: activity, enumeration, isolation and characterization of thiosulfate-oxidizing bacteria. Syst Appl Microbiol.

[b41-ehp-118-1183] Stein ED, Cohen Y, Winer AM (1996). Environmental distribution and transformation of mercury compounds. Crit Rev Environ Sci Technol.

[b42-ehp-118-1183] Ullrich SM, Tanton TW, Abdrashitova SA (2001). Mercury in the aquatic environment: a review of factors affecting methylation. Crit Rev Environ Sci Technol.

[b43-ehp-118-1183] U.S. EPA (U.S. Environmental Protection Agency) (1997). Mercury Study Report to Congress. An Assessment of Exposure to Mercury in the United States.

[b44-ehp-118-1183] U.S. EPA (U.S. Environmental Protection Agency) (2001a). Method 1630: Methyl Mercury in Water by Distillation, Aqueous Ethylation, Purge and Trap, and CVAFS.

[b45-ehp-118-1183] U.S. EPA (U.S. Environmental Protection Agency) (2001b). Water Quality Criterion for the Protection of Human Health: Methylmercury.

[b46-ehp-118-1183] U.S. EPA (U.S. Environmental Protection Agency) (2002). Mercury in Water by Oxidation, Purge and Trap, and Cold Vapor Atomic Fluorescence Spectrometry (Method 1631, Revision E).

[b47-ehp-118-1183] Wang SF, Feng XB, Qiu GL, Fu XW, Wei ZQ (2007). Characteristics of mercury exchange flux between soil and air in the heavily air-polluted area, eastern Guizhou, China. Atmos Environ.

[b48-ehp-118-1183] WHO. (World Health Organization) (1990). Methylmercury. Environmental Health Criteria 101.

[b49-ehp-118-1183] WHO. (World Health Organization) (1991). Inorganic Mercury. Environmental Health Criteria 118.

[b50-ehp-118-1183] WHO (World Health Organization) (2003). Concise International Chemical Assessment Document 50. Elemental Mercury and Inorganic Mercury Compounds: Human Health Aspects.

[b51-ehp-118-1183] Zhang H, Feng X, Larssen T, Shang L, Vogt RD, Rothenberg SE (2010). Fractionation, distribution and transport of mercury in rivers and tributaries around Wanshan Hg mining district, Guizhou province, southwestern China. Part 1. Total mercury. Appl Geochem.

